# Sterilized *Anopheles funestus* can autodisseminate sufficient pyriproxyfen to the breeding habitat under semi-field settings

**DOI:** 10.1186/s12936-023-04699-9

**Published:** 2023-09-21

**Authors:** Hamisi J. Kunambi, Halfan Ngowo, Ali Ali, Naomi Urio, Amos J. Ngonzi, Yohana A. Mwalugelo, Mohamed Jumanne, Augustino Mmbaga, Felista S. Tarimo, Joseph Swilla, Fredros Okumu, Dickson Lwetoijera

**Affiliations:** 1 Environmental Health and Ecological Science Department, Ifakara Health Institute, P.O. Box 53, Ifakara, Tanzania; 2 School of Life Science and Bio-Engineering, The Nelson Mandela African Institution of Science and Technology, P.O. Box 447, Arusha, Tanzania; 3 Tanzania Biotech Products Limited, The National Development Cooperation, P.O. Box 30119, Kibaha, Tanzania; 4 School of Public of Health, Faculty of Health Science, University of Witwatersrand, Johannesburg, South Africa; 5 Institute of Biodiversity, Animal Health and, Comparative Medicine, University of Glasgow, Glasgow, G12 8QQ UK; 6 Department of Biomedical Sciences, Jaramogi Oginga Odinga University of Science and Technology, P.O. Box 210-40601, Bondo, Kenya

**Keywords:** *Anopheles funestus*, Autodissemination, Pyriproxyfen, Sterilization, Semi-field settings

## Abstract

**Background:**

*Anopheles funestus*, the main malaria vector, prefer to oviposit in permanent and/or semi-permanent breeding habitats located far from human dwellings. Difficulties in identifying and accessing these habitats jeopardize the feasibility of conventional larviciding. In this way, a semi-field study was conducted to assess the potential of autodissemination of pyriproxyfen (PPF) by *An. funestus* for its control.

**Methods:**

The study was conducted inside a semi-field system (SFS). Therein, two identical separate chambers, the treatment chamber with a PPF-treated clay pot (0.25 g AI), and the control chamber with an untreated clay pot. In both chambers, one artificial breeding habitat made of a plastic basin with one litre of water was provided. Three hundred blood-fed female *An. funestus* aged 5–9 days were held inside untreated and treated clay pots for 30 min and 48 h before being released for oviposition. The impact of PPF on adult emergence, fecundity, and fertility through autodissemination and sterilization effects were assessed by comparing the treatment with its appropriate control group.

**Results:**

Mean (95% CI) percentage of adult emergence was 15.5% (14.9–16.1%) and 70.3% (69–71%) in the PPF and control chamber for females exposed for 30 min (*p* < 0.001); and 19% (12–28%) and 95% (88–98%) in the PPF and control chamber for females exposed for 48 h *(p* < 0.001) respectively. Eggs laid by exposed mosquitoes and their hatch rate were significantly reduced compared to unexposed mosquitoes (*p* < 0.001). Approximately, 90% of females exposed for 48 h retained abnormal ovarian follicles and only 42% in females exposed for 30 min.

**Conclusion:**

The study demonstrated sterilization and adult emergence inhibition via autodissemination of PPF by *An. funestus.* Also, it offers proof that sterilized *An. funestus* can transfer PPF to prevent adult emergence at breeding habitats. These findings warrant further assessment of the autodissemination of PPF in controlling wild population of *An. funestus*, and highlights its potential for complementing long-lasting insecticidal nets.

## Background

Long-lasting insecticidal nets (LLINs) and indoor spray with residual insecticides (IRS) have remained the core interventions for malaria control [1, 2]. However, the gains achieved with these indoor-based interventions are threatened by the on-going development of insecticide resistance within targeted malaria vector populations [3, 4]. Worryingly, increased outdoor biting as a result of mosquito behavioural adaptation, and change in human behaviour by spending more time outdoors altogether makes these tools less effective in sustaining the gains [5–9]. Despite the urgent call for additional vector control tools to complement LLINs and IRS to accelerate the efforts toward malaria elimination, the additional tools need to align with the local context of the specific malaria endemic countries [10].

Larval source management (LSM) particularly chemical or microbial larviciding, is one of the promising tools that can be used in conjunction with adult-based interventions for effective control of malaria vectors [11–13]. Larviciding has additional benefits because it targets the aquatic stage of vectors and thus controls the population of exophilic, endophagic, and resistant mosquitoes that are associated with malaria transmission [13, 14]. It is recommended by the World Health Organization (WHO) to be implemented as a supplementary intervention in areas where breeding habitats are only few, findable, and easy to map and treat [15].

The potential of using larviciding for malaria control in urban settings was first demonstrated in Tanzania as part of urban malaria control programme, resulting to a 32% reduction in annual mean vector densities and sporozoite prevalence in three malaria vectors: *Anopheles funestus*, *Anopheles coustani* and *Anopheles gambiae* [16]. The government of Tanzania, leveraged these successes, and established the Tanzania Biotech Products Limited (TBPL), a bio-larvicide plant. This initiative has enabled the piloting of the larviciding intervention to 11 municipals in 2016 and gradually scaled up to all municipal councils in 2020 [17–19].

Effective implementation of larviciding depends on the accurate identification and targeting of productive mosquito breeding habitats [20]. This necessitates a sustainable surveillance system for monitoring the availability and distribution of breeding habitats before implementation [21–23]. Recently, the application of geospatial technology and deployment of unmanned aerial vehicles (UAV) have proven effective in identifying and targeting water bodies in a wide space, which would not have been possible by only relying on personnel [24–26]. On the other hand, both UAV and geospatial technology are resource demanding, and require high operational and analysis skills. In addition, they are both limited in distinguishing the mosquito productive breeding habitats from merely water bodies [25, 26]. On this basis, the high cost and limitations associated with both the conventional larviciding and deployment of UAV highlight the need for alternative larviciding strategies that are cost-effective and complementary to LLINs and IRS, such as the autodissemination technique [27–29].

The autodissemination technique, also known as “Mosquito-assisted larviciding” is a technique that exploit females mosquito oviposition behaviour, to transfer insecticides from a contaminated resting station to its breeding habitat and results in mortality or prevents emergence of the immature mosquito therein [28]. Several studies have demonstrated the effectiveness of the autodissemination technique, mainly with pyriproxyfen, an insect growth regulator, in controlling *Aedes*, *Culex*, and *Anopheles* mosquitoes under controlled and field settings [28–34]. The autodissemination technique interrupts malaria transmission by preventing the emergence of adult vectors from contaminated breeding habitats resulting in the reduction of malaria vector density [35, 36]. By relying on female mosquitoes that know suitable places to breed, this technique can precisely enhance high coverage of targeted breeding habitat during field application and overcome the need for widespread application of insecticide and excessive use of labour [32, 37, 38].

Pyriproxyfen a juvenile hormone mimic is an insect growth regulator (IGR) that has been demonstrated to effectively control disease-carrying mosquitoes of different species [29, 39–41]. Pyriproxyfen works by mimicking the action of a naturally occurring juvenile hormone by interfering with the growth and development of the target insect resulting in either sterilizing the contaminated mosquitoes [42, 43] or inhibiting adult emergence [44]. In addition, the compound is highly specific and effective at a ultralow concentration [45]. To date, there is no evidence of pyriproxyfen resistance in malaria vectors with practical implications, with exception of animal model experiments that suggest pyriproxyfen can be metabolized in the same way as pyrethroid [46]. Another study highlighted a partial increase in the level of mosquito tolerance to pyrethroids when used in sub-lethal doses [47].

Of importance, pyriproxyfen is the safe compound, with allowable amount of 300 parts per billion in human drinking water, which is 6 times higher than amount recommended by the WHO for effective mosquito control [45]. While the autodissemination technique and sterilization impact of pyriproxyfen has been demonstrated with *An. gambiae* and *An. arabiensis* [29, 34], the ability of *An. funestus*, a dominant malaria vector, to perform autodissemination of pyriproxyfen remains unknown. Therefore, this study was conducted in a semi-field setting in rural Tanzania to evaluate the efficacy of pyriproxyfen to control the *An. funestus* via autodissemination and sterilization effects.

## Methods

### Study site

The study was conducted at Kining’ina village (8.11417 S, 36.67484 E), in rural southern Tanzania from May to October 2022 inside a partitioned netted cage with dimensions of 6 m long, 2.25 m wide, 2 m high, built within the semi-field system. The partitioned, netted cage made with fine mesh was installed inside the semi-field chamber to prevent *An. funestus* from escaping upon release, due to its small body size [48, 49].

### Mosquitoes rearing

Insectary-reared female *An. funestus* aged 5–9 days post eclosion were used for the experiments. These colonies originated from individual wild *An. funestus* mosquitoes collected from three villages (Tulizamoyo, Ikwambi and Sululu) within the Kilombero valley in 2019. The standardized rearing procedures of the *An. funestus* mosquito colonies has already been described [48]. It has been reported that the insemination rate increases as the age of male mosquitoes increases [50]. Therefore, it was considered essential to mix a batch of 5–9 days old females with male *An. funestus* of older age, 10–14 days in the same cages to increase the chance of mating. Female mosquitoes were starved for 12 h prior to blood feeding, by placing arm of consented human inside the cage for 30 min. These procedures were repeated on two consecutive days to ensure that the mosquitoes were fully engorged.

### Test insecticide

Sumilarv dust containing pyriproxyfen of 10% active ingredient and 12 μm particle size, supplied Sumitomo Chemicals, Japan, was used in all experimental replicates.

### Preparation of contamination station

In all experimental replicates, a clay pot of 10-litre volume was used as a contamination station. It was prepared by lining the inside with damp black cotton cloth dusted with 2.5 g of 10% pyriproxyfen powder using a paint brush [29]. Prior to mosquito exposure, the dusted clay pot was left to dry for 24 h to allow the pyriproxyfen powder to attach to the cotton fibers and enhance pick-up by female mosquitoes upon landing.

### Experimental procedures and study design

#### **Experiment 1: assessment of pyriproxyfen transfer from pyriproxyfen-treated clay pot to the breeding habitat by female *Anopheles funestus***

Two chambers of the semi-field system which were 1.5 m apart were used as the treatment and control chambers for this experiment. In the treatment chamber, a PPF-dusted clay pot and a breeding container of 1 L of water were installed at a distance of 5 m apart. Two experiments (30 min and 48 h exposure) of five replicates each were performed by exposing mosquitoes to PPF inside the clay pot. In each replicate, a batch of 300 blood-fed mosquitoes were separately held inside treated clay pot for 30 min and 48 h before were released for oviposition. The breeding habitat container was monitored for the presence of eggs for two consecutive days before being removed from the chamber.

Because the number of eggs that were naturally deposited in the breeding containers were few, the presence of PPF was confirmed via larval bioassay, during which 20 instar three insectary-reared larvae of *An. funestus* were introduced in a container that was removed from the chamber, and monitored for daily mortality and emergence success until all were dead or emerged to adults. A similar setup was adopted for the control chamber.

#### **Experiment 2: assessment of fertility, reduction in egg laying, and eggs viability as a proxy indicator for pyriproxyfen sterilization effect on exposed female *Anopheles funestus***

Few numbers of eggs signaled the possibility of sterilization effect to exposed females. Hence, the sterilizing effect of PPF on exposed *An. funestus* via pyriproxyfen autodissemination was assessed using a sub sample of mosquitoes from experiment 1 (60 mosquitoes per replicate). The samples were gently aspirated on day three post their first blood meal. Of 60 mosquitoes, 30 were dissected for examination of ovary development, and 30 were transferred into a separate net cage (15 × 15 × 15 cm) and monitored for oviposition events over three gonotrophic cycles.

Prior dissection, *An. funestus* females were anesthetized by freezing at − 20^°^C for 10 min. Mosquitoes were dissected by gently pulling out the last two segments of the abdomen under the stereoscopic microscope at 0.7x magnification and the extracted ovaries were further observed under a compound microscope for a focused view of ovary appearance. The ovaries’ development status was recorded as normal when they appeared fully developed at stage V or previtellogenic resting stage and abnormal when underdeveloped at IV. The classification and interpretation of ovary appearance were based on the Christopher stage of egg development [51].

During oviposition monitoring, an oviposition substrate made of a petri dish and wet/damp cotton lined with filter paper was placed inside the net cage. The *An. funestus* were maintained with 10% glucose solution *ad libitum*. The oviposition substrate was then followed up for a maximum of five days to observe the presence of eggs. If the eggs were spotted, then the filter paper containing the eggs was removed and eggs were counted and transferred into a 300 ml plastic cup filled with tap water. Therein, eggs were monitored for daily hatching for up to seven days consecutive until all eggs hatched to larvae or died [42, 52, 53].

Following the first egg-laying cycle, the remaining *An. funestus* were offered a second blood meal to assess the effect of PPF on the second gonotrophic cycle. Three days post blood feeding, the oviposition substrate was supplied for egg laying. Similar monitoring procedures for egg development and hatching as described above were followed. Following the second egg-laying cycle, similar procedures were repeated to assess the effect of PPF on the third gonotrophic cycle [54].

### Statistical analysis

Statistical software, R version 4.2.1, was used to analyze the data. Generalized linear mixed-effects models (GLMM) were implemented using functions within the lme4 package [55]. The difference in the total number of laid eggs between the control and treatment chambers was determined with Poisson distribution using the best fit Generalized linear mixed model. The difference in the adult emergence rate of third instar larvae previously introduced into water basins was determined with a binomial GLMM using the logit function. The difference in the total number of eggs laid by the batch of female mosquitoes in the net cage and their hatch rate was also analysed using Poisson and binomial GLMM, respectively. In the model, the experiment chamber (with or without PPF) was classified as a fixed effect, whereas the experiment replicate was classified as a random effect variable. In addition, Percentage reduction of fertility per gonotrophic cycle was calculated using the following formulation. Reduction in fertility (%) per gonotrophic cycle = (C − T)/ C × 100 where C = hatch rate in the unexposed group; T = hatch rate in the exposed group.

## Results

### **Pyriproxyfen transfer from pyriproxyfen-treated clay pot to the breeding habitat by female *Anopheles funestus***

Both exposure times (30 min and 48 h) resulted in the transfer of pyriproxyfen from the PPF-treated clay pot to the provided breeding habitats. In all five experimental replicates, at 30 min of exposure, the mean (95% CI) percentage of adult emergence was 70% (69–71%) in the control chamber compared to 15.5% (14.916.1%) in the treatment chamber (*p* < 0.001). Similarly, at 48 h of exposure, the mean (95% CI) percentage of adult emergence was 95% (88 – 98%) in the control chamber compared to 19% (12 –28%) in the treatment chamber (*p* < 0.001) (Table 1). The significantly lower percentage of adult emergence in the treatment chamber suggest that provided breeding habitats were contaminated with pyriproxyfen by ovipositing female *An. funestus*.

**Table 1:** Percentage of adult emerged from the third instar larvae in the breeding habitats

Exposure time	Section	Proportion % [95% CI]	OR [95% CI]	*P*-values
30 min	Control	70.3 [69.3, 71.2]	1	< 0.001
	Pyriproxyfen	15.5 [14.9, 16.1]	0.07 [0.03, 0.21]	
48 h	Control	95.0 [88.4, 97.9]	1	< 0.001
	Pyriproxyfen	18.9 [12.2, 28.2]	0.01 [0.004, 0.03]	

*OR* odds ratio, *CI* confidence interval, *min* minutes, *hrs* hours

**Table 2:** Mean number of larvae hatched from laid eggs by *An. funestus* in the control and pyriproxyfen-contaminated breeding habitats

Exposure time	Section	Predicted mean [95% CI]	RR [95% CI]	*P*-value
30 min	Control	189.7 [139.6, 257.9]	1	< 0.001
	Pyriproxyfen	26.7 [19.4, 36.8]	0.14 [0.13, 0.16]	
48 h	Control	249.4 [218.9, 284.1]	1	< 0.001
	Pyriproxyfen	49.6 [42.9, 57.4]	0.20 [0.18, 0.22]	

*RR* relative risk, *CI* confidence interval, *min* minutes, *hrs* hours

### **Effect of autodissemination of pyriproxyfen on the *Anopheles funestus *reproductive capacity**

As summarized in Table 2, female mosquitoes contaminated with pyriproxyfen laid significantly fewer eggs (estimated from recorded larvae) compared to the uncontaminated mosquitoes in the control chamber (*p* < 0.001). For 30 min and 48 h exposure, the mean number (95%CI) of mosquito larvae resulting from laid eggs in the control chamber were 89.7 ± 25.56 and 249.5 ± 15.56 respectively. The number of larvae in their respective treatment groups were significantly low, 26.7 ± 3.72 for 30 min and 49.6 ± 3.42 for 48 h exposure (*p* < 0.001).

**Fig. 1:**
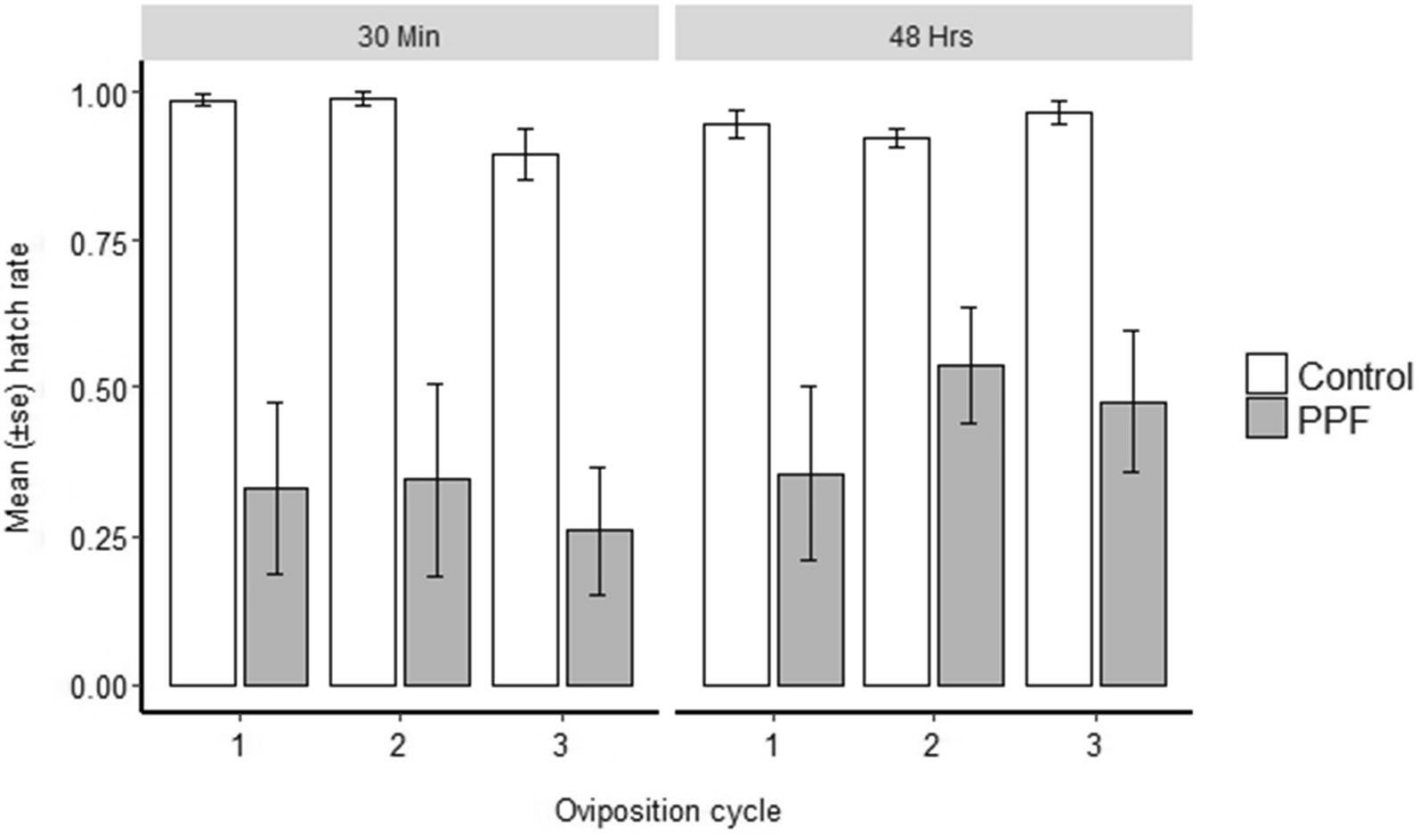
The hatch rate of the eggs laid by exposed and unexposed female *Anopheles funestus* across three oviposition cycles

Compared to the control group, the hatch rate was significantly lower in the treatment group across all three gonotrophic cycles for both exposure times (*p* < 0.001) (Fig. 1). In a treatment group, hatchability decreases with increase in gonotrophic cycles for both exposure times. Overall, the reduction of hatch rate at 30 min exposure was 15.4%, 52.9%, and 60.6% for the first, second, and third gonotrophic cycles respectively, whereas at 48 h exposure were 20.8%, 45% and 51.5%, respectively.

### **Effect of pyriproxyfen exposure on female *Anopheles funestus *sterility/fertility**

**Fig. 2:**
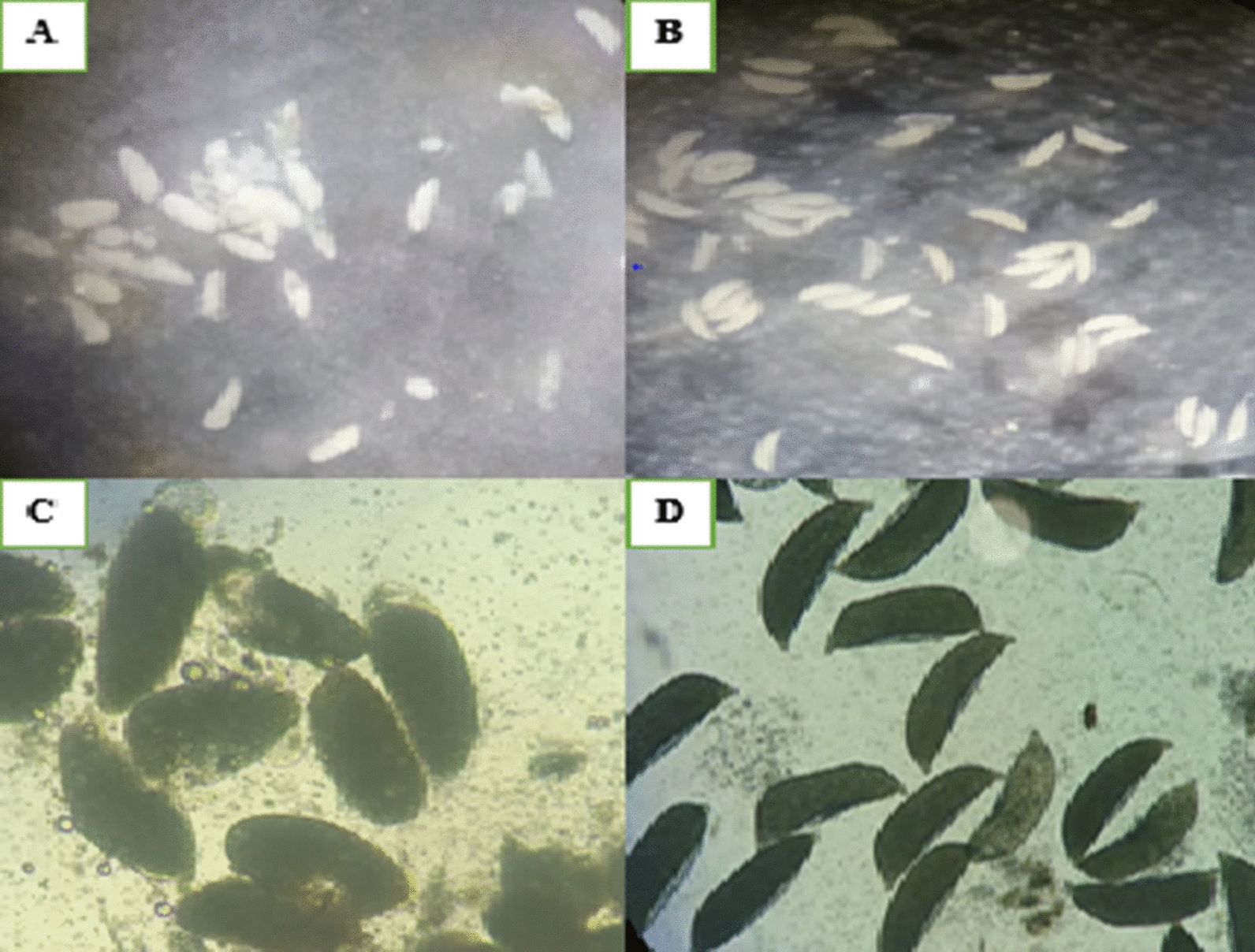
Morphological features of an eggs of PPF-exposed and unexposed blood-fed female *Anopheles funestus* at 72 h post blood meal. **A** and **C** showing observed abnormal eggs (undetachable, oval shape with no floats). **B** and **D** showing observed normal eggs (detachable, boat/sausage shape with floats)

Pyriproxyfen exposure adversely affected female mosquitoes eggs development (Fig. 2; Table 3). Of the 590 exposed and unexposed female *An. funestus* that were dissected to observe the effect of PPF on eggs development, 35% (n = 205) had abnormal eggs, and the remaining 65% (n = 385) had normal eggs. During 30 min exposure, 4% (n = 6) of 141 females in a control group had abnormal eggs compared to 42% (n = 63) of 149 in the treatment group. Whereas for 48 h exposure, 4% (n = 6) of 150 females in a a control group had abnormal eggs compared to 87% (n = 130) of 150 females in the treatment group. However, normal eggs of the females exposed for 48 h were observed to have fewer abnormal eggs arrested at stage IV.

**Table 3:** Effect of pyriproxyfen exposure on female* An. funestu*s fertility

Effect of pyriproxyfen on *An. funestus* fertility	30 min Exposure		48 h Exposure	
	PPF	Control	PPF	Control
Mosquito exposed	1500	1500	1500	1500
Mosquitoes dissected	149	141	150	150
Percentage of sterilized females (with 100% abnormal eggs)	42%	4%	87%	4%
Percentage of fertile females	58%	96%	13%	96%

*PPF* treatment chamber, *min* minutes, *hrs* hours

## Discussion

The current study has proven that sterilized female *An. funestus* exposed one day post blood meal, can transfer a lethal dose of pyriproxyfen to the breeding habitat located 5 m from a contaminated clay pot. Overall, forced contaminated *An. funestus* with pyriproxyfen resulted to 78% and 81% adult emergence inhibition of its filial at 30 min and 48 h of exposure respectively. These findings are corroborated by previous studies in *Anopheles* that documented successful autodissemination events by *An. arabiensis*, *An. gambiae* and *Anopheles quadrimaculatus*, via either self or forceful mosquito contamination [29, 31, 33, 34, 56]. The recorded similarity in emergence inhibition at 30 min and 48 h might be due to loss of the picked pyriproxyfen particles because of their grooming behaviour when mosquitoes are exposed longer, its absorption to mosquito cuticle, and during flight to breeding habitat [57–59].

In this study, the females were held in presence of pyriproxyfen for 30 min and 48 h to mimic possible minimum and maximum resting time for rest seeking blood fed mosquitoes in the field environment [60–64]. In a situation where mosquitoes are transiting the contamination stations, the success of autodissemination events might be impaired [28, 57, 65].

Of importance, this study documented *An. funestus* vulnerability to pyriproxyfen sterilization after being exposed one day post blood meal, and confirmed significant reduction in eggs laid (fecundity). Overall, at 30 min and 48 h of pyriproxyfen exposure, the mean number of eggs laid by the exposed group was reduced by 85.9% and 80.1% respectively compared to the control group. Similarly, negative effect of pyriproxyfen on mosquito fecundity has been also shown in several studies [34, 42, 54, 66, 67]. Consistent with previous study [54], the effect of pyriproxyfen on fecundity and fertility (eggs hatchability) in exposed *An. funestus* was observed up to third gonotrophic cycle, suggesting that this effect might be irreversible during mosquito lifespan.

Previous studies have reported that pyriproxyfen interferes with the balance of hormones levels between juvenile hormone and ecdysone hormone, and disrupt the hormonal pathways responsible for egg’s maturation [44, 54, 66, 68]. Similarly, in this currently study, the dissection of PPF exposed female mosquito revealed that pyriproxyfen sterilization effect was via retention of under developed (unmatured) eggs. Longer exposure time resulted to high proportion of mosquitoes that retain underdeveloped eggs compared to shorter exposure time. Many underdeveloped eggs were arrested at Christopher stage IV, a proxy indication for sterilization effect [68]. It has been documented in other studies that the sterilization effect interferes with the desire of contaminated female to find a place for oviposition. [34, 58, 66]. This depends on the time of pyriproxyfen exposure relative to when the female obtains a blood meal. While Mbare and others reported unlikelihood of contaminated female mosquito to visit the oviposition habitat after being exposed to pyriproxyfen within 24 h before and after the blood meal [34], Itoh et al., reported the frequency of visiting the oviposition habitat to be lower for female exposed to pyriproxyfen before blood meal and higher for female exposed to pyriproxyfen after blood meal [58].

Furthermore, Yadav et al. [66], when assessing surface treated with a range of pyriproxyfen concentrations, reported a lower frequency of visiting oviposition habitat to a female exposed to a lower concentration of pyriproxyfen at 24 h before blood meal and higher to the females exposed at 24 h after blood. But the frequency of visiting the oviposition habitat was the same only for the female exposed to higher concentration [66]. In this current study, female mosquitoes exposed 24 h post blood meal were capable of visiting oviposition habitat. The difference in oviposition behaviour for contaminated female mosquitoes across different studies might be due to differences in pyriproxyfen exposure methods, pyriproxyfen formulation (e.g., powder or suspension), pyriproxyfen doses and environments under which the study was conducted.

It has been reported in previous studies that environmental factors, such as wind speed, temperature, and relative humidity are responsible for triggering oviposition flights of gravid female mosquitoes [69]. Because the current study was conducted in a semi-field environment, the observed oviposition behaviour in sterilized *An. funestus* is more representative to what might happen under actual field settings compared to similar studies that were conducted under laboratory conditions [34, 58, 66].

Overall, the findings of this study further support the potential of autodissemination of pyriproxyfen in controlling primary susceptible and resistant malaria vectors. More striking, is the fact that sterilized mosquitoes were capable to autodisseminate pyriproxyfen enough to cause adult emergence inhibition at the breeding habitats. Therefore, its potential use could be aligned with the current recommended integrated vectors control approach, which focuses on controlling and eliminating outdoor and residual malaria transmission [15, 70, 71]. Furthermore, this presents an opportunity of scaling up this technique along recently recommended next generation bed nets co-treated with pyriproxyfen and pyrethroid [71]. It was envisaged that, host-seeking resistant mosquitoes sterilized by pyriproxyfen nets might transfer pyriproxyfen upon successful access to a bloodmeal and resting in a contaminated station. In addition, the combined effect of these two modes of actions of pyriproxyfen can be mathematically modelled to assess its additive or synergistic effect on malaria transmission interruption.

The appropriate time for deploying autodissemination of pyriproxyfen is mainly during the dry season [72, 73] characterized by few but stable breeding habitats. This season provides ideal condition to attain optimal doses to prevent adult emergence in the breeding habitats. On the contrary, implementing autodissemination of pyriproxyfen during rainy season, associated with flooding, hence dilution of PPF in the breeding habitats, might amplify resistance levels of the already pyrethroid resistant mosquito population as the results of the sub-lethal doses in the habitats [47].

Despite achieving the main objective of this study, some limitations were observed. The effect of autodissemination of pyriproxyfen was not directly monitored at the provided breeding habitat but through larval bioassays. All experiment were conducted in presence of small water volumes (1 L), which was important to prove the principle, but not representative of actual habitats found in the field environment [74]. Lastly, the resistance status of the exposed mosquitoes was not assessed, instead the supposition that they were resistant was based on the most recent reports from the same study area [75–78].

## Conclusion

The present study demonstrates sterilization and adult emergence inhibition effects in *An. funestus* via autodissemination of pyriproxyfen. It also offers proof that sterilized *An. funestus* can transfer pyriproxyfen sufficient to prevent adult emergence at its habitats. These promising findings warrant further assessment of the autodissemination of pyriproxyfen in controlling wild population of *An. funestus*, and emphasize its potential in complementing LLINs.

## Data Availability

All data underlying the presented results in this study will be available upon request.

## Abbreviations

AI: Active ingredient
GTS: Global technical strategy
IRS: Indoor residual spray
IGR: Insecticide growth regulator
LSM: Larval source management
LC: Lethal concentration
LLINs: Long Lasting Insecticidal nets
NMSP: National Malaria Strategic Plan
SFS: Semi field system
PPF: Pyriproxyfen
TBPL: Tanzania Biotechnology Product Limited
WHO: World Health Organization
